# Validation of heart failure diagnosis registered in primary care records in two primary care centres in Barcelona (Spain) and factors related. A cross-sectional study

**DOI:** 10.1080/13814788.2017.1305104

**Published:** 2017-04-05

**Authors:** Jose María Verdú-Rotellar, Eva Frigola-Capell, Rosa Alvarez-Pérez, Daniela da Silva, Cristina Enjuanes, Mar Domingo, Amparo Mena, Miguel-Angel Muñoz

**Affiliations:** ^a^ Centro de Atención Primaria Sant Martí de Provençals, Institut Català de la SalutBarcelonaSpain; ^b^ Institut d’Investigació en Atenció Primaria IDIAP-Jordi GolBarcelonaSpain; ^c^ Departament de Medicina, Facultat de Medicina, Universitat Autónoma de BarcelonaBellaterraSpain; ^d^ Programa Integrado de Atención a la Insuficiencia Cardiaca del Área Integral de Salud Barcelona Litoral Mar, Servei Català de la SalutBarcelonaSpain; ^e^ Parc de Salut MAR, Servicio de Cardiologıa, Hospital del MarBarcelonaSpain; ^f^ Centro de Atención Primaria Sant Roc, Institut Català de la SalutBadalonaSpain; ^g^ Centro de Atención Primaria Congres, Institut Català de la SalutBarcelonaSpain; ^h^ Departament de Obstericia, Ginecologia i Medicina Preventiva, Facultat de Medicina, Universidad Autónoma de BarcelonaBellaterraSpain

**Keywords:** Heart failure, primary health care, accuracy of diagnosis

## Abstract

**Background:** Heart failure (HF) diagnosis as reported in primary care medical records is not always properly confirmed and could result in over-registration.

**Objectives:** To determine the proportion of registered HF that can be confirmed with information from primary care medical records and to analyse related factors.

**Methods:** A cross-sectional study. The medical records of 595 HF patients attended in two primary healthcare centres in Barcelona (Spain) were revised and validated by a team of experts who classified diagnosis into confirmed, unconfirmed, and misdiagnosis. Variables potentially related to the confirmation of the diagnosis were analysed. The revision of medical records and data collection took place from 15 January to 31 March 2014.

**Results:** Mean (standard deviation) age was 78 (10) years and 58% were women. The diagnosis could be confirmed in 53.6% of patients. Factors associated with a greater probability of having a confirmed diagnosis were age (yearly OR: 0.97, 95%CI: 0.95–0.99), cardiologist follow-up (OR: 3.66, 95%CI: 2.46–5.48), history of ischaemic heart disease (OR: 2.18, 95%CI: 1.36–2.48), atrial fibrillation (OR: 2.01, 95%CI: 1.34–3.03), and prescription of loop diuretics (OR: 3.24, 95%CI: 2.14–4.89).

**Conclusion:** Only in half of the patients labelled as HF in primary care medical records could this diagnosis be further confirmed. Variables regularly registered in clinical practice could help general practitioners identify those patients requiring a revision of their HF diagnosis.

KEY MESSAGES

- Heart failure diagnosis is not always properly classified in primary care medical records.
- Variables regularly registered in the clinical records of patients with heart failure could help general practitioners who suspect that the diagnosis has to be revised.

## Introduction

Heart failure (HF) is a major public health concern. Its incidence increases with age from 1% in individuals aged less than 50 years to 8% in those over 75 [1]. Recent population-based studies conducted in Spain have shown a prevalence of 4% to 7%, which can reach up to 16% in elderly patients [2,3].

The role of general practitioners (GPs) in the initial diagnosis and management of patients with HF has been well documented, particularly in elderly populations with comorbidity and polypharmacy [3–6]. Diagnosis requires demonstrating cardiac dysfunction and remains a challenge: HF signs and symptoms are not specific and in many cases difficult to identify [1,3,5,7–11]. It comprises a wide range of patients from those with HF with preserved ejection fraction (HF-PEF) to those with reduced ejection fraction (HF-REF) with LEVF <40%. Differentiation of patients with HF based on LVEF is important due to distinct underlying aetiologies, demographics, comorbidities, and therapy response [1].

Even though HF diagnosis requires demonstrating cardiac dysfunction, it has been reported that 20% to 40% of HF patients had not had an echocardiogram registered in their primary care medical records [4,6,12]. Based on these data, in previous studies [11,13–17], HF diagnosis had been confirmed in 35% to 85% of the registered cases. To date, evidence regarding the factors associated with a confirmed diagnosis of HF is scarce [18–20].

It has also been recently argued that a well-documented previous hospital admission because of HF could be considered as proof of properly identified diagnosis [2,3,5,21].

The aim of this study was to determine the proportion of registered HF, which can be confirmed with information from primary care medical records and to analyse the related factors.

## Methods

### Study design

A cross-sectional study was carried out in two primary care centres (PCC) located in Barcelona, which provide healthcare to 39 000 individuals. The medical records of all patients registered with an HF diagnosis were reviewed. Since both PCCs belong to the Catalan Health Institute, they use the same electronic medical record system. In computerized medical records, information on symptoms, examination, comorbidities, complementary tests, and patient referrals is systematically collected.

An integrated healthcare programme in which cardiologists provide support to GPs by sharing consultations and training programmes is also included in this system [22].

### Ethics

This study complies with the Declaration of Helsinki. Ethical approval was obtained from the Health Care Ethics Committee of the Institut d'Investigació en Atenció Primária Jordi Gol (reference number P14/021).

### Study population and inclusion criteria

#### Inclusion criteria

All patients aged >14 years registered on 1 January 2014 with the diagnostic code I.50 (I.50.0: congestive HF, I.50.1: left ventricular HF) according to the International Classification of Diseases (tenth revision) were included.

#### Exclusion criteria

Deceased patients and those who had moved to other PCCs were excluded, since updated information was not available.

#### Study period

The revision of clinical records and data collection took place from 15 January to 31 March 2014.

### Confirmatory diagnosis of HF

A reference panel consisting of a specifically trained GP and a cardiologist classified patients into confirmed, unconfirmed, and misdiagnosis, according to the following criteria:

1. Confirmed HF (at least one of the following):
1.1. Presence in the primary care medical records of typical HF signs and symptoms (dyspnoea, orthopnoea, paroxysmal nocturnal dyspnoea, oedema, elevated jugular venous pressure, third heart sound) along with an echocardiography showing structural anomalies of HF according to the European Society of Cardiology [1,9].
1.2. The existence of an echocardiography showing structural anomalies of HF in the primary care electronic medical records without typical HF signs and symptoms (asymptomatic HF) [1,9].
1.3. Information about a hospitalization because of HF [23].
2. Misdiagnosis of HF was considered if:
2.1 Typical HF signs and symptoms are registration in the primary care medical records but echocardiography did not show structural abnormalities.
2.2. Neither registration of typical HF signs and symptoms nor echocardiography showed structural abnormalities.
3. Unconfirmed diagnosis was considered if echocardiography had not been performed or information about the procedure was unavailable.

### Variables

The research team reviewed the medical records of the patients included. To confirm the primary outcome (confirmatory diagnosis of HF), they collected the variables needed to establish the diagnosis of HF: typical HF signs and symptoms, left echocardiographic findings (ventricular ejection fraction, abnormalities of diastolic relaxation, or left atrial enlargement, or left ventricular hypertrophy, and or moderate or severe valve disease), and the previous hospitalization as a consequence of HF was also taken into account.

To analyse the variables related with confirmed HF, data related to the diagnosis of HF in the literature were collected in addition to information from the electronic medical record system (Table 1) such as loop diuretic prescription, demographic characteristics, date of HF diagnosis, and whether patients were seeing a cardiologist [1,19,20]. The following comorbidities were analysed: hypercholesterolaemia, hypertension, diabetes mellitus, ischaemic heart disease, chronic obstructive pulmonary disease, asthma, chronic kidney disease, smoking, obesity, atrial fibrillation, ischaemic heart diseases.

**Table 1.** Demographic and clinical characteristics according to the presence of a confirmed diagnosis of heart failure.

			Heart failure not confirmed (*n* = 276) *n* (%)		
	Total population (*n* = 595) *n* (%)	Heart failure confirmed (*n* = 319) *n* (%)	Misdiagnosis *n* = 226	Unconfirmed *n* = 50	*p*
Demographic variables					
Age (mean, SD)	78.3 (10.5)	77.3 (11.2)	79.63 (8.1)	78.96(10.7)	.02
Sex (women)	346 (58.1)	169 (52.9)	145 (64.1)	32 (65.2)	.05
Cardiovascular risk factors					
Body mass index (mean, SD)	29.87 (5.2)	29.5 (5.2)	30.2 (5.4)	30.2 (4.5)	.52
Hypertension	499 (83.9)	260 (81.5)	201 (8.9)	38 (76)	.038
Diabetes mellitus	291 (48.9)	154 (48.3)	121 (53.5)	16 (32)	.69
Hypercholesterolaemia	377 (63.4)	208 (65.2)	143 (63.3)	26 (52)	.56
Smoking	45 (7.6)	29 (9.1)	14 (6.2)	2 (4)	.09
Cardiovascular comorbidities					
Ischaemic heart disease	165 (27.7)	121 (37.9)	39 (17.2)	5 (10)	<.01
Atrial fibrillation	236 (39.7)	161 (50.4)	61 (26.9)	14 (28)	<.01
No cardiovascular comorbidities					
Chronic kidney disease	152 (25.5)	93 (29.5)	46 (20.3)	13 (26)	.04
Chronic obstructive pulmonary disease	158 (26.5)	102 (31.9)	45 (19.9)	11 (22)	.01
Asthma	30 (5.0)	14 (4.4)	15 (6.6)	1 (2)	.58
Other variables					
Visits by cardiologist	319 (53.6)	227 (71.1)	89 (39.4)	3 (6)	<.01
ECG normal	36 (6.1)	18 (5.6)	17 (7.5)	1 (0.2)	.65
Loop diuretics	321 (53.9)	207 (64.9)	114 (50.4)	12 (24)	<.01
Time since onset (years) [interquartile range]	4 [3–8]	4 [3–8]	4 [3–8]		.9

**Table 2.** Univariable and multivariable analysis and confirmed diagnosis of heart failure.

	Univariate analysis		Multivariate analysis^a^	
	OR (95%CI)	*p*	OR (95%CI)	*p*
Demographic variables				
Age	0.96 (0.94–0.99)^b^	.01	0.97 (0.95–0.99)	.04
Sex (women)	1.07 (0.58–1.95)	.82	0.74 (0.49–1.13)	.16
Cardiovascular risk factors				
Body mass index	0.91 (0.92–1.02)	.21		
Hypertension	1.03 (0.52–2.05)	.92		
Diabetes mellitus	1.23 (0.74–2.05)	.40		
Hypercholesterolaemia	0.93 (0.56–1.55)	.80		
Smoking	0.71 (0.36–1.39)	.32		
Cardiovascular comorbidities				
Ischaemic heart disease	1.76 (1.03–3.18)	.04	2.17 (1.36–3.48)	.01
Atrial fibrillation	1.68 (1.01–2.78)	.04	2.01 (1.34–30.3)	.01
No cardiovascular comorbidities				
Chronic kidney disease	1.16 (0.67–2.00)	.58		
Chronic obstructive pulmonary disease	1.37 (0.74–2.54)	.32		
Asthma	1.39 (0.50–3.90)	.53		
Other variables				
Visits by cardiologist	4.83 (2.92–7.99)	<.01	3.66 (2.46–5.47)	<.01
ECG normal	0.52 (0.11–2.16	.44		
Loop diuretics	3.48 (2.07–5.84)	<.01	3.23 (2.14–4.89)	<.01
Time since onset	0.99 (0.93–1.05)	.77		

^a^ Multivariate analysis has been adjusted for age, sex and variables statistically significant at the univariate.

^b^ Age: OR per year.

### Statistical analysis

For data analysis, diagnosis of HF was categorized into confirmed and not confirmed (unconfirmed and misdiagnosis). Descriptive data of factors potentially associated with the diagnosis are presented. For categorical variables, frequencies were reported. For continuous variables, mean and standard deviation (SD) were calculated. Median and interquartile range (IQR) were assessed for the variable ‘time since HF onset,’ which did not follow normal distribution after using the Kolmogorov–Smirnov test. Chi-square and Student–Fisher tests for categorical and continuous variables, respectively, were employed for bivariate analyses. Variables significantly (*p* <.05) associated with the primary outcome in the bivariate analysis were included as potential covariates in logistic regression models. Forward and backward step techniques with the likelihood ratio test were employed. Multivariate adjusted odds ratios and 95% confidence intervals were calculated. All analyses were performed with SPSS Inc. v17.0 software.

## Results

**Figure 1.**
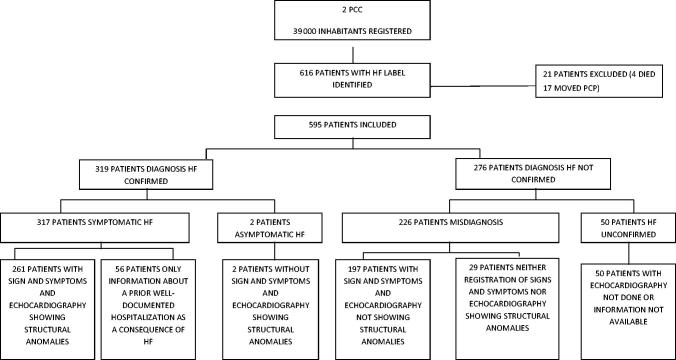
Validation of heart failure diagnosis. PCC: primary care centres, HF: heart failure.

Initially, 616 potentially eligible patients with an HF diagnosis in their primary care electronic medical records were identified. Twenty-one were excluded because they either had moved to another PCC or had died before the validation process (Figure 1). The validation of HF diagnosis was finally carried out in 595 patients. Mean (standard deviation) age was 78 (10) years and 58% were women. Registered prevalence of HF was 1.5%. At least one echocardiography was found in 558 medical records (93.8%). In 13 (2.2%) patients, the echocardiography had been performed but information was unavailable.

In 319 (53.6%) patients, the diagnosis of HF was confirmed after the revision. Of the 276 (46.4%) patients with not confirmed HF, 226 were a misdiagnosis, and 50 unconfirmed (Figure 1). Demographic and clinical characteristics of the patients are shown in Table 1.

Among patients with confirmed HF, 32.1% had left ventricular ejection fraction <50%, 37.8% had abnormalities of diastolic relaxation, 30.7% left atrial enlargement, 21.0% left ventricular hypertrophy, and 5.0% moderate or severe valve disease.

Multivariate regression models showed that the factors associated with a higher probability of having confirmed diagnosis of HF were age (yearly OR: 0.97, 95%CI: 0.95–0.99), cardiologist follow-up (OR: 3.66, 95%CI: 2.46–5.48), history of ischaemic heart disease (OR: 2.18, 95%CI: 1.36–2.48), atrial fibrillation (OR: 2.01, 95%CI: 1.34–3.03), and prescription of loop diuretics (OR: 3.24, 95%CI: 2.14–4.89) (Table 2).

## Discussion

### Main findings

In this study, we found that it was possible to confirm further HF diagnosis in only half of the patients labelled as such in the primary care medical records.

Patients with cardiologist follow-up, and those with a history of ischaemic heart disease, atrial fibrillation, and receiving loop diuretics, are more likely to have a confirmed HF diagnosis.

### Strengths and limitations

To confirm diagnostic accuracy, a systematic protocol agreement was established for both GPs and cardiologists.

Information was collected retrospectively on diagnosis. As a result, it is possible that some signs and symptoms were not registered in the patient’s medical records.

Previous research has demonstrated that, in the primary care setting, the use of NT-proBNP in the assessment of patients with possible HF increases diagnostic accuracy [24]. Unfortunately, natriuretic peptide determination is not available in our daily practice.

The main limitation of the study could be that it was carried out, for reasons of feasibility, in only two PCCs, which are also involved in protocols with cardiologists and this could affect the external validity of the study. Another limitation is that, unfortunately, we do not have data that allow us to assess the variability of the diagnosis according to the characteristics of the GPs.

### Interpretation of the study: Results in relation to existing literature

The registered prevalence of HF in our study (1.5%) was greater than in other previously reported studies using a similar methodology [25], and a high percentage of patients in our sample were found to have had an echocardiography registered in their medical records. This could be because for several years the participating PCCs have taken part in an integrated HF care programme involving specialized and primary care sharing management protocols. This kind of experience has shown to be successful in the diagnosis and management of HF patients [26,27].

A recent meta-analysis reported that more than 85% of HF diagnoses could be confirmed through the data available in the administrative database [28]. However, most studies in this meta-analysis used clinical criteria as the gold standard to confirm the diagnosis and were hospital-based. A study carried out in primary care found that only in 34% of patients labelled as HF in primary care medical records could diagnosis be confirmed [29].

The elevated number of patients labelled as HF and having a normal echocardiogram is noteworthy. It is possible that their echocardiography had been performed months or years after the diagnosis was recorded.

In Europe, it is frequent to find HF diagnosis in general practice without confirmatory tests [5,13].

The proportion of patients with no evidence of signs or symptoms agrees with other studies where it appears that GPs are less likely to record them than the results of complementary tests [7,10,28].

Regarding the association of morbidity related to confirmed diagnosis, it is known that atrial fibrillation is associated with HF-PEF and that coronary artery disease is the origin of two-thirds of systolic HF [24]. Previous studies had found a high probability of HF misdiagnosis when a history of coronary heart disease or atrial fibrillation was not present [19,20].

The association of HF diagnosis with the prescription of diuretics has also been described [10,21]. It is probable that patients with symptoms due to volume load are often treated with loop diuretics. In fact, the latest European Society of Cardiology guidelines included the use of this medication as a suspected HF factor [29]. Nevertheless, at least one consultation with the cardiologist is recommended to confirm the initial diagnosis [27,30].

We believe that our findings could be extrapolated to other countries with health systems and populations similar to ours. The variables which we have found related to confirmed HF are usually collected in primary care and should not differ amongst other European countries [31,32].

### Implications for clinical practice

The diagnosis of HF in the medical records of the patient is an important label that conditions treatments, tests, and interventions. The accuracy of diagnosis, however, remains a challenge: HF signs and symptoms are not specific, and in many cases difficult to identify. Diagnosis requires demonstrating cardiac dysfunction.

The data from our work support the need to perform periodic self-audit in patients registered with HF. It should be prioritized in those patients who do not meet criteria to confirm diagnosis, performing the necessary examinations or additional tests. Once patients are re-classified, databases should be adjusted, removing the registry of HF in those patients in whom diagnosis has been ruled out after a correct revision. In this way, unnecessary interventions and treatments can be avoided. The results of our study also support, especially in case of doubt, the need for the collaboration of the cardiologist in confirming diagnosis.

## Conclusion

Only in half of the patients labelled as heart failure in primary care medical records could this diagnosis be further confirmed.

Variables regularly registered in clinical practice records could help general practitioners identify those patients requiring a revision of their heart failure diagnosis.
